# Review on Fluorescent Carbon/Graphene Quantum Dots: Promising Material for Energy Storage and Next-Generation Light-Emitting Diodes

**DOI:** 10.3390/ma15227888

**Published:** 2022-11-08

**Authors:** Ashish Gaurav, Amrita Jain, Santosh Kumar Tripathi

**Affiliations:** 1 Graduate Institute of Photonics and Optoelectronics, National Taiwan University, Taipei 10617, Taiwan; 2 Institute of Fundamental Technological Research, Polish Academy of Sciences, Pawińskiego 5B, 02-106 Warsaw, Poland; 3 Department of Physics, School of Physical Sciences, Mahatma Gandhi Central University, Motihari 845401, Bihar, India

**Keywords:** carbon dots, graphene quantum dots, white-LED, supercapacitors, Na-ion batteries, Li-ion batteries

## Abstract

Carbon/graphene quantum dots are 0D fluorescent carbon materials with sizes ranging from 2 nm to around 50 nm, with some attractive properties and diverse applications. Different synthesis routes, bandgap variation, higher stability, low toxicity with tunable emission, and the variation of physical and chemical properties with change in size have drawn immense attention to its potential application in different optoelectronics-based materials, especially advanced light-emitting diodes and energy storage devices. WLEDs are a strong candidate for the future of solid-state lighting due to their higher luminance and luminous efficiency. High-performance batteries play an important part in terms of energy saving and storage. In this review article, the authors provide a comparative analysis of recent and ongoing advances in synthesis (top-down and bottom-up), properties, and wide applications in different kinds of next-generation light-emitting diodes such as WLEDs, and energy storage devices such as batteries (Li-B, Na-B) and supercapacitors. Furthermore, they discuss the potential applications and progress of carbon dots in battery applications such as electrode materials. The authors also summarise the developmental stages and challenges in the existing field, the state-of-the-art of carbon/graphene quantum dots, and the potential and possible solutions for the same.

## 1. Introduction

**Figure 1:**
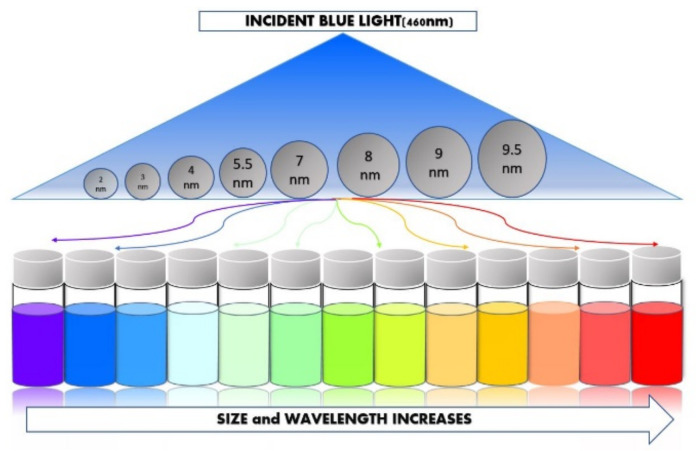
Different sized quantum dots [6].

In recent years, nanotechnology has emerged as a boom in the scientific world and has become a base and pillar for development and research in various scientific fields such as healthcare, energy storage, energy-conversion optoelectronics, sensors, and detection. A large number of studies have been carried out in the field of nanotechnology and are still ongoing; one is the evolution of quantum dots (QD) and their advancements, which provide numerous opportunities to examine their application in the diverse field of the basis of its unique properties such as quantum confinement, size-tunable wavelength, and broad excitation range. QDs can emit light when excited by UV light, comprehensive and continuous absorption spectra, and narrow emission with high light stability [1,2,3]. QDs can be defined as semiconductor inorganic crystals that contain a plethora of electrons occupying well-defined and discrete quantum states [4]. Their promising fluorescent properties are due to their small size, which provides a wide range of variable element ratios [5]. They are also known as artificial atoms producing discrete energy levels, whose band gap can be tuned by varying their sizes. Figure 1 shows the relationship between the energy band gap with quantum dot particle size, which is inverse in nature [6]. QDs absorb white light and re-emit specific colours within the range of nanoseconds depending on the band gap of the material [3].

In the past few years, quantum dots have been widely explored by researchers across the globe in different fields, such as tracking [7], cell imaging [8], light-emitting diodes [9], energy storage [10], and many others. Along with the renewables-based lithium/sodium ion batteries, metal-air batteries, supercapacitors, fuel cells, and water-splitting technologies, the use of quantum dots and their derivatives have also been explored for energy storage and conversion applications [11,12,13].

In the field of energy storage, lithium-ion batteries have exciting and well-known features such as a long lifespan and high energy density, due to which they have shown great applications ranging from electrifying vehicles to transferable electronics. However, the restricted supply of lithium metal (because of the chronic shortage of lithium salts) limits the use of lithium-ion batteries to a great extent [14]. This increases motivation to find new materials that can replace lithium—one potential solution is sodium salts, which have the potential to replace lithium-ion batteries because of the high availability and low cost of sodium. Hence, sodium-ion batteries have been explored and studied, but they have many disadvantages, such as low capacity, poor cyclic durability, and inferior rate capability, because of the larger ionic size of sodium, which narrows its practical use [15].

Therefore, developing novel materials with favourable properties to meet the world’s requirements without harming the environment is a crucial challenge for researchers to overcome existing complications. The prime focus and inspiration of researchers across the globe are to draft new ideas and innovations to develop more environmentally friendly materials that can be prepared using existing abundant natural resources. One of the promising outcomes is the development of quantum dots by easy and\or facile techniques using carbon-containing materials available in nature.

**Figure 2:**
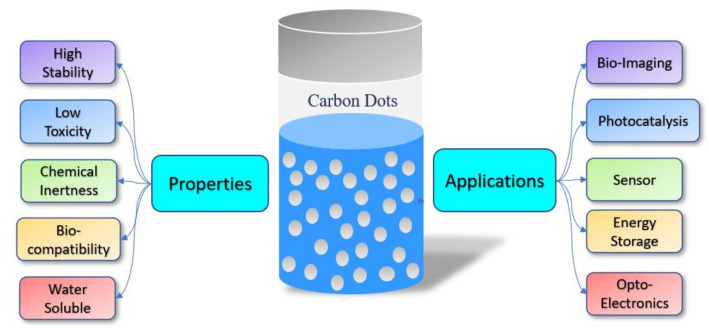
CQD properties and applications [16].

Due to the wide adoption in various applications and the intense need for QDs, this review article mainly focuses on carbon/graphene-derived QDs and their potential applications in energy storage and bio-imaging. Carbon quantum dots (CQD) are defined as quasi-spherical carbon nanoparticles with ultrafine sizes under or around 10 nm [16]. Due to their various properties (Figure 2), such as easy synthesis, high stability, good bio-compatibility, low toxicity, robust chemical inertness, excellent photostability, and high water dispersibility, CQDs have great potential in the field of wastewater treatment [17,18], detection of heavy metal ions [19], and high contrast bio-imaging and bio-sensing application such as in drug delivery [20], supercapacitors [21], and solar cells [22].

The oxygenated functional group on the surface of CQDs remodifies the structure and particle size of the CQD, which results in the quantum confinement effect [23]. It is worth mentioning that the fluorescent properties of CQD are greatly affected by the emissive property of the carbon precursor used for the fabrication of CQD [24]. Initially, raw materials used for CQD production included carbon-containing sugar, citric acid, and carbohydrates due to their complex organic structures [25]. Furthermore, due to the large π- conjugated electronic structure and size-dependent quantum confinement effect, CQD can be used in energy storage devices as transport media [26] and as anode material for potassium-ion batteries [27]. In belonging to the family of zero-dimensional (0D) nanoparticles, graphene quantum dots (GQD) have also been a subject of research due to their unique properties such as solid quantum confinement and edge effects of QDs such as photoluminescence, high conductivity, chemical inertness, high stability, environment friendly [28,29,30], bio-compatible, dissolvable in organic solvents, and high electron mobility [31]. Furthermore, they consist of highly crystalline and very thin graphene with sp^2^ hybridised carbon dimensions < 100 nm. Additionally, from density functional theory, it has been found that the band gap of GQD with individual aromatic rings reached around 7.4 eV [32]. As a result of properties such as good conductivity, large specific surface area, and ease of doping and structural modification, GQD can further add power as a conductive agent to the electrode in energy storage devices such as supercapacitors. One of the interesting fluorescent properties related to GQD compared to other semiconductor quantum dots (QD) is that as the excitation wavelength increases, the luminescent peak broadens and is red-shifted, resulting in fluorescent characteristics depending on the excitation wavelength [33]. The eye-catching luminescent properties of GQD make them a strong candidate for their development in the biological field, such as bio-imaging and bio-sensors [34].

**Figure 3:**
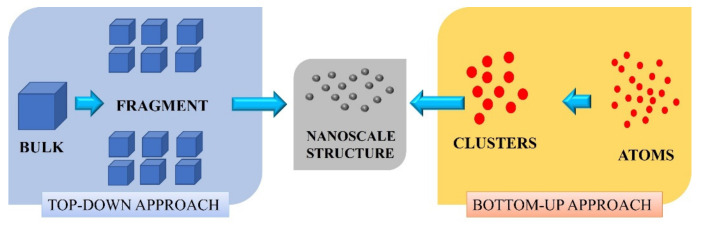
Top-down and bottom-up techniques [28].

To date, various methods have been adopted and developed for the fabrication of carbon quantum dots (CQD) and graphene quantum dots (GQD). Top-down and bottom-up are the two standard techniques for fabricating quantum dots [28] (Figure 3).

In the top-down methods, CQD are synthesised via breaking down carbon-containing materials such as graphene, carbon fibres, nano-diamonds, carbon soot, and other carbon sources. On the other hand, in a bottom-up approach, the integration of various aromatic compounds and complex chemical compounds, such as citric acid, amino acid, and carbohydrates, results in the formation of CQD [35,36]. Currently, various preparation methods for different CQD/GQDs have been favoured, which include hydrothermal, chemical oxidation, electrochemical etching, laser-induced, microwave-assisted, ultra-sonication, solvothermal, electron beam irradiation, stripping, etc. Among them, the hydrothermal method is widely preferred because it uses cheap, eco-friendly biomass components and is easy to handle compared to other methods. Among these various pathways, researchers have closely monitored and altered various parameters during fabrication to obtain CQD/GQD with abundant physical and optical properties for their applications in different fields [37,38,39,40].

## 2. Methodology

**Figure 4:**
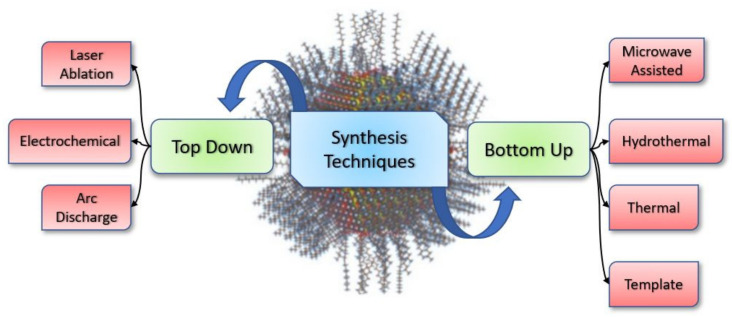
Various synthesis routes for CQD/GQDs [42].

To date, a plethora of methods has been adopted for the effective synthesis of carbon/graphene quantum dots or carbon dots. Various methods can be divided into two groups: top-down and bottom-up approaches. With the inadvertent discovery of fluorescent carbon-based nanoparticles [41], the top-down approach played an important role in forming CQDs for many years, which was further overtaken by the bottom-up approach because of easy and less strident synthesis conditions. Initially, the quantum yield of fluorescent nanoparticles based on carbon obtained by the path of laser ablation followed by oxidation and surface passivation was around 10% [42]. In the top-down approach, the CQDs are derived from the disengagement of large carbon source precursors such as carbon nanotubes [41], graphite rod/powder [43], carbon fibres, or even carbon soot [44]. Some of the properties of carbon-containing macromolecules, such as ample amounts of sp^2^ carbon, an infinite Bohr diameter and the absence of effective band gap, which can produce luminescence on excitation, are the key reason for adopting the top-down methods, as splitting these large carbon sources into nano-scale particles will result in efficient PL through quantum confinement effects [45]. Some of the standard top-down techniques include arc discharge [46] and electrochemical and laser ablation (Figure 4) [42,47].

Whereas, in the bottom-up approach, dehydration, polymerisation, and carbonisations of various small molecules result in CQD/GQD by the route of different chemical reactions. Microwave-assisted, hydrothermal processes, thermal processes, and template processes are some of the standard methods followed by researchers. Furthermore, carbon sources and experimental conditions (e.g., change in temperature, reaction time, and altering the ratio of precursors) dramatically affects the size and optical properties of the carbon dots formed using different routes.

## 3. Top-Down Approach

### 3.1. Laser Ablation

**Figure 5:**
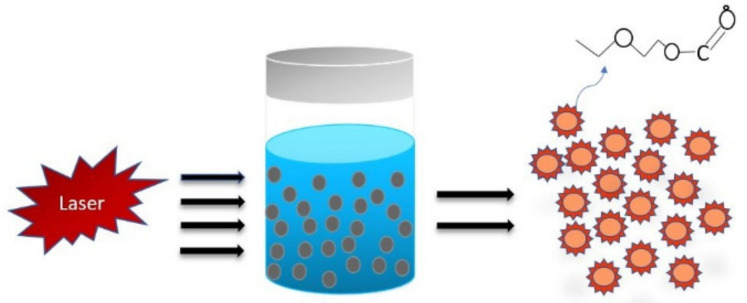
One-step synthesis of carbon nanoparticles in PEG200N solvent using laser ablation [43].

In this method, the synthesis of highly fluorescent carbon dots is achieved by laser irradiation on different carbon sources. It is a method in which high energy laser pulse is used to illuminate the surface of the carbon source to a thermodynamic state where high pressure along with the high temperature is created, which further heats up and vaporises into a plasma state followed by vapour crystallisation resulting in nanoparticles [48,49]. Initially, CQDs were fabricated via laser ablation of the carbon source with a tide of argon gas in the presence of water vapour at a temperature of 900 °C and a pressure of 75 kPa. As a result, carbon dots were formed in an agglomerated form with almost no (or less) detectable PL. Then, fusing the aqueous solution of HNO_3_ with the formed sample followed by refluxing and passivating the surface by adhering organic species such as PEG_1500N_ (amine-terminated polyethylene glycol) (200 mg, 0.13 mmol at 120 °C for 72 h) and PPEI-EI, i.e., poly(propionylethyleneimine-co-ethyleneimine) resulted in highly luminescent CQDs with a size around 5 nm and at the excitation at 400 nm, the quantum yield was 4% to 10% [42]. Synthesis of fluorescent carbon dots using Nd:YAG pulsed laser with a wavelength of 1.064 µm and a power density of 6 × 10^6^ W cm^−2^ was used to illuminate the graphite powder, which acted as the carbon source suspended in different solvents, and it was observed that the nature of the solvent played an essential role in the size of carbon dots formed (Figure 5) [43]. 

Another researcher used a small amount of nano-carbon material and dispersed it into 50 mL of different solvents such as ethanol, acetone, and water. After ultrasonication, 4 mL of solution was dropped on a glass substrate/cell covered by a quartz window for laser illumination with Nd:YAG pulsed laser [50]. One study reported the fabrication of CQD using graphitic particles ranging a few micrometres. The source is dipped in water, and a pulse laser of wavelength 1070 nm and pulse duration of 50 ns was implemented. With continued stirring to avoid precipitation, the heat energy caused by the laser resulted in the disintegration of the carbon source into tiny particles [18,51].

Using this technique, different structure nanoparticles can be prepared, and carbon particles can be constructed using ethanol via laser ablation [52]. Laser illumination/irradiation helps form different carbon nanoparticles, but the carbon nanoparticle yield is low in this technique compared to other techniques [53].

### 3.2. Electrochemical Methods

**Figure 6:**
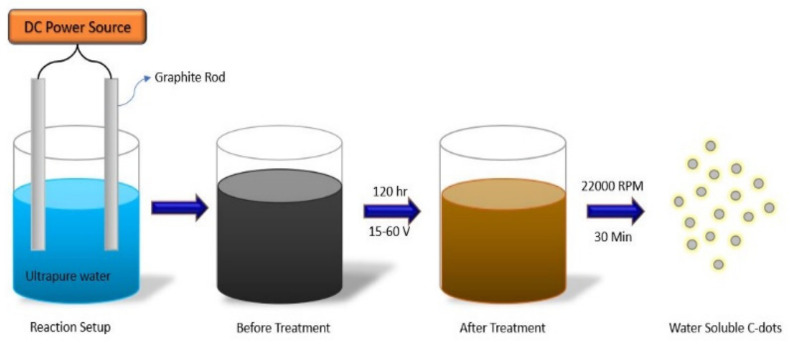
CQD using electrochemical reaction in ultrapure water [59].

In all the various techniques used to fabricate CQDs, issues such as size controllable formation of C-dots for investigating the intrinsic PL properties are yet to be solved. Many researchers have reported size control by changing parameters such as growth temperature, reactant, surfactant concentration, and surfactant concentration [54]. Among the various methods to date, the electrochemical approach helps control the size significantly by regulating the current density and applied electrode potential. This method is widely preferred because it is easy to alter the particle size and PL performance of the so-formed CQD [55,56]. An oversimplified way in which electrochemical carbonisation of low-molecular-weight alcohols is by taking the alcohol as the precursor and further converting it into carbon-containing particles after electrochemical carbonisation under normal conditions; then, the size can be varied just by changing the applied potential [57]. Another report suggested that the preparation of graphene quantum dots or graphene oxide quantum dots by the electrochemical exfoliation pathway—in which two graphitic rods are used as electrodes dipped in an electrolyte composed of citric acid and alkali hydroxide in water by varying one parameter (e.g., concentration of alkali hydroxide) while keeping the other parameters constant—resulted in different types of GQD formations [56]. Monodispersed C-dots can be further obtained by electrochemical etching of carbon fibres in acetonitrile containing tetrabutylammonium perchlorate (TBAP) by adjusting the applied potential without further surface passivation [58]. The synthesis of high-purity and quality carbon dots has been proposed using a one-step electrochemical pathway in pure water with no assistance from different chemical compounds. A pure graphite rod (99.99%) was dipped into a beaker containing ultrapure water as an anode (18.4 MΩ cm^−1^, 600 mL) with a separation of 7.5 cm with its counter electrode placed parallelly in the solution. By applying 15–60 V using a DC power supply and stirring the solution for two hours, the graphite rod corroded with an appearance of dark yellow solution in the setup. Furthermore, the solution was filtered using quantitative filter paper, which was followed by centrifugation of the solution at 22,000 rpm for 30 min resulting in the separation of graphite oxide and graphite particles and as a final product, water solution C-dots were obtained as shown in Figure 6. Another reported the formation of monodispersed C-dots with a diameter ranging from 5–8 nm by chemical oxidation treatment of flour [59,60].

### 3.3. Arc Discharge

The simplicity, ability for natural phase separation, and scalability in production features of the arc discharge method have drawn huge attention in recent years. Carbon dots with a low quantum yield (QY) of 1.6% have been synthesised using crude carbon nanotube soot. The soot was oxidised with 3.3 M HNO_3_, and then the oxidised material was extracted with an alkaline solution of pH 8.4, after which the material was purified by conducting gel electrophoresis [61,62]. Recently, one study reported the synthesis of fluorescent carbon dots using the submerged arc discharge in water (SADW) method. The whole process was performed in a rectangular stainless-steel chamber. Graphitic rods were used as an anode and cathode of various diameters. Electrical arc discharge was performed in distilled water. Generally, in SADW, the product after arc discharge is separated into three different forms: floating, suspended, and precipitate. The suspended material in water is primarily composed of CQDs and a few graphene oxides [63,64].

## 4. Bottom-Up Approach

### 4.1. Microwave-Assisted Technique

**Figure 7:**
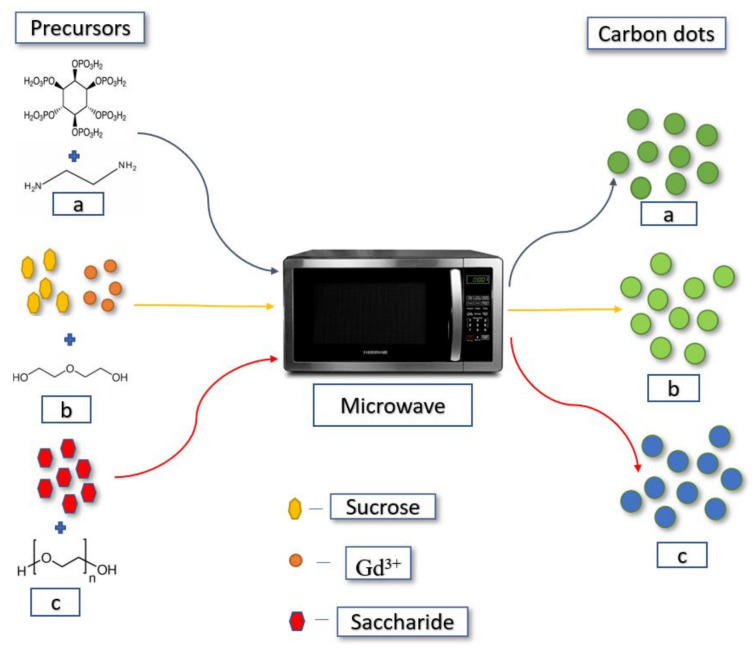
CQD synthesis using the microwave assistive method [72,73,74].

The microwave-assisted technique is the most effective method because of its high reaction rate and short time consumption [65]. The low-cost, efficient, and facile technique for the formation of CQD shows a disadvantage of low quantum yield (<10%) in most cases [66,67,68]. Implementing this method on organic compounds such as saccharides results in a low-cost route for the synthesis of CQDs (Figure 7c) [69,70]. Many researchers have reported CQD formation using this simple technique, which goes under polymerisation and carbonisation [71]. Water-soluble phosphorus-containing carbon quantum dots were synthesised using this facile technique by using phytic acid as the carbon source and ethylenediamine in ultrapure water, which resulted in strong green fluorescent CQDs (Figure 7a) [72]. Carbon nitride dots of size around 4.5 nm with a high quantum yield of 18.9% were reported with blue fluorescence and are dispersible in water using microwave-assisted polyol using the folic acid molecule as both a carbon and nitrogen precursor [73]. Another researcher synthesised gadolinium-doped carbon dots using various chemicals such as diethylene glycol, sucrose, and Gd_2_O_3_ via a one-step microwave-assisted polyol method. The so-formed Gd-CQD displayed green emission with a low quantum yield of 5.4% and showed evidence of low toxicity (Figure 7b) [74]. With a PLQY of around 30.2%, carbon dots have been synthesised using citric acid as a carbon source, containing a carboxyl group to facilitate the dehydration and carbonisation and 1,2-ethylenediamine as a surface passivating agent using the microwave pyrolysis technique [69,75]. 

Using poly(ethylene glycol) PEG as a precursor and passivating agent, C-dots were synthesised, and being photostable and low cytotoxicity showed some promising results for bio-imaging and biolabeling [76]. CQDs with an average particle diameter of 10.14 nm for cancer-imaging applications were synthesised using waste cotton linter as a carbon source via a microwave-assisted hydrothermal process [77]. Another report suggested CQD synthesis using lignocellulosic residue from the pulp and paper industry via a microwave-assisted reaction catalysed by a solid acid catalyst, which promoted the formation of CQD [78]. With less than 60 s of reaction time, CQD was synthesised using phthalic acid and triethylenediamine hexahydrate as precursors through this facile microwave-assisted method [79]. Recently, mono-dispersed carbon quantum dots (CQD) with excellent photoluminescence were manufactured via microwave plasma-enhanced decomposition (MPED) using fenugreek seeds, which acted as a natural carbon precursor. The formation of CQD using the MPED method was 97.2% faster than any other traditional thermal decomposition method [80].

### 4.2. Hydrothermal/Solvothermal

Application of an aqueous medium over 100 °C and 0.1 MPa is generally known as a hydrothermal condition because most minerals are found in this environment [81]. In recent years, the hydrothermal process has been widely accepted for the synthesis of new solids along with diverse kinds of inorganic material, for example, functional oxide and non-oxide nanomaterial with specific size and shape configurations [82,83]. Generally, the hydrothermal process is of two types based on the formation temperature. The high-temperature hydrothermal process operates in the range of 300 to 800 °C, whereas the low-temperature hydrothermal process happens below 300 °C.

**Figure 8:**
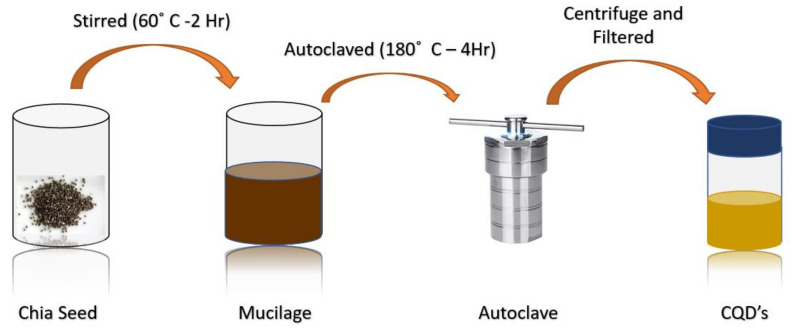
CQD synthesis via the hydrothermal method [86].

For carbon-based materials, by changing the solubility, melting the crystalline parts, promoting acid/base and ionic reactions, and accelerating the physical and chemical interaction between the reagents, which results in the formation of carbon-based structures and materials, the hydrothermal process is widely preferred [84]. Treating glucose with monopotassium phosphate dissolved in DI water via a hydrothermal approach resulted in the formation and surface passivated highly fluorescent CQDs whose emission can be tuned by altering the concentration of the monopotassium phosphate [66]. With a high quantum yield of 80%, carbogenic carbon dots were synthesised using citric acid and ethylenediamine using this hydrothermal process [67]. Another reported the formation of nitrogen-doped carbon quantum dots and showed blue emission with promising properties such as excellent stability in high-salt conditions, great photostability, and a quantum yield of 84.79% using citric acid and diethylenetriamine in one pot via this technique [68]. For ion-detection and cell imaging applications, nitrogen-doped CQDs (N-CQD) were formed using glucose and phenylenediamine through the hydrothermal process and acted as an excellent fluorescent probe for Fe^3+^ and CrO_4_^2−^ and cell imaging reagent for Hela cells [85]. In recent years, green chemistry has created a new pathway for the production of nanoparticles at a larger scale using various kinds of natural carbon sources. Recently, C-dots were synthesised using 1 gm chia seed in 100 mL of distilled water and then heated until the solution reached a temperature of 60 °C. The obtained mucilage was then taken to a Teflon-based autoclave at 180 °C for 4 hr. The formed solution was further sonicated for 30 min, followed by centrifugation at 10,000 rpm for 15 min, which resulted in the isolation of larger particles; further repeating the process for various steps leads to the formation of a brown transparent solution containing CQDs (Figure 8) [86]. 

**Figure 9:**
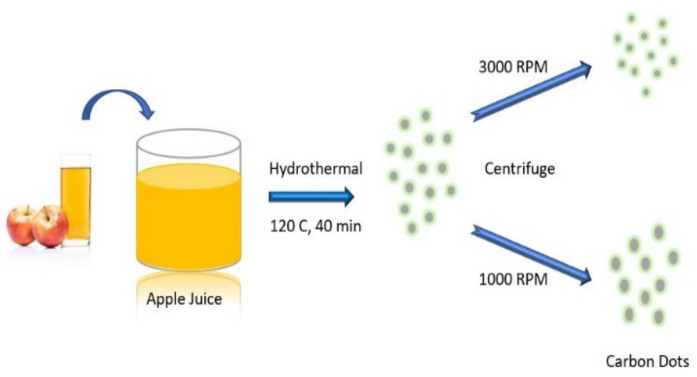
CQD using apple juice via hydrothermal route [90].

Xie et al. [87] reported a one-step hydrothermal synthesis of nitrogen and silicon co-doped carbon quantum dots with high product yield (52.56%) and high quantum yield (97.32%) using citric acid monohydrate and silane coupling agent KH-792. Full-colour range CQD with highly controllable fluorescence has been fabricated using citric acid and urea in formamide in two steps involving synthesis and separation [88]. Sulphur-doped graphene quantum dots using durian as a carbon source in a platinum catalyst has been reported with good optical and chemical stabilities along with ultrahigh quantum yield [89]. Another interesting advantage of the hydrothermal method is its freedom of choice of the precursor, such as juices, citric acid, and glucose can be widely used for hydrothermal synthesis. Recently, carbon dots have been reported using apple juice via a hydrothermal process in which 40 mL of apple juice was mixed with 30 mL of ethanol; the mixture further went to autoclave at 120 °C for 40 min. The formed brown solution was taken for centrifugation, and the obtained filtered solution was mixed with 40 mL of chloroform in a funnel and stirred for separation. Then, the separated solution was centrifuged at different rpm speeds. Initially, it was centrifuged at 3000 rpm, and the material with low fluorescence was isolated; 20 mL of acetone was added to the left-over sample and was further centrifuged. As a result, carbon dots with sizes ranging from 4 to 10 nm were obtained with high fluorescence properties (Figure 9) [90].

### 4.3. Thermal/Combustion Method

In recent years, the combustion route has gained significant attention due to its easy scaling, higher control over designing the starting molecules, and environmentally friendly features. Multicolour fluorescent carbon nanoparticles with size <2 nm and water-soluble were synthesised from the combustion of soot/sediment of candles. Treating the soot/sediment with an oxidant such as HNO_3_, H_2_O_2_/AcOH resulted in the formation of carbon dots. Detached using polyacrylamide gel electrophoresis, the formed carbon dots were found to be highly mobile with PL at shorter emission wavelength [44,61]. Another report suggested the preparation of GQD with a uniform particle size of 8.5 nm by combustion of citric acid at 200 °C succeeded by functionalisation with a carboxyl group under high temperature [91,92].

### 4.4. Template Method

The template method is rarely used by researchers for the fabrication of nano-size carbon quantum dots. This method has two levels: (a) the development of carbon dots through calcination in a suitable mesoporous silicon sphere or template and (b) etching to create C dots in the nano range by erasing the support, but the etching route is complicated due to the requirement of higher-temperature, difficulty in purification, and limited quantum yield (QY) [75,93,94]. Photoluminescent mono-dispersed with uniform morphology carbon dots having tunable sizes, compositions, and other properties were synthesised using four different carbon sources as precursors via a soft-hard template approach [75]. Zong et al. [95] prepared carbon dots ranging 1.5–2.5 nm using mesoporous silica spheres and citric acid as hard templates and carbon sources, respectively. The size-controlled soft template approach for the synthesis of oleylamine carbon dots has been reported with carbon dots acting as a photoactive material. The emulsifier played a crucial role in varying the size of carbon dots [96]. 

For the top-down approach, many reports have shown an immense amount of control over the morphology and the size of the CQDs formed. Along with controllable dimensions, methods such as electrochemical oxidation result in higher purity and yield with good reproducibility. Other top-down techniques help in large-scale productions with easy operational routes in the case of chemical oxidation or the ultrasonic route. In contrast, laser ablation and electrochemical methods have complicated operational mechanisms with higher costs, limiting their wider acceptance.

For the Bottom-Up approach, techniques like microwave synthesis have been very promising in terms of reaction time and uniformity in size with easily controllable size fabrication, whereas hydrothermal treatments are non-toxic and highly efficient with higher quantum efficiency in material synthesis. In contrast to the pros, hydrothermal treatment has the disadvantage of lower production yield [50,97,98].

## 5. Characterisation: Physical and Chemical Properties

With different techniques for forming CQDs with a goal of desirable properties for various useful applications, various techniques have been widely adopted to characterise the formed carbon dots using different synthesising approaches. Some of the essential characterisation tools for physical and optical properties include transmission electron microscopy or high-resolution transmission electron microscopy (TEM/HRTEM) to approximate the size of the CQD/GQD formed, X-ray diffraction (XRD) to check the different phase formation in carbon dots, Fourier transform infrared spectroscopy (FTIR) to review and analyse the bonding between various compounds and elements in the carbon dots, UV-visible spectroscopy (UV-vis) to estimate the band gap, absorption of the synthesised CQD, photoluminescence spectroscopy (PL) for the emissive property of various CQD/GQDs, and many other characterisations have been used till date as per the requirements.

### 5.1. Optical Properties

#### 5.1.1. UV-Absorption

CQD prepared by different techniques shows strong UV absorption, and the absorption peak’s position varies with the method adopted to synthesise the CQD. Mostly, they show optical absorption in the ultraviolet region, extending a little in the visible range. These peaks can be attributed to the π-π* transition of sp^2^ conjugated carbon or C=C bonds and n-π* transition of hybridisation with heteroatoms such as N, P, S, or the transition of C=O bonds [91,99]. One report suggested that the dependency of absorption range strongly depends on size, as with the increase in size, the absorption peak shifts from the UV range to the NIR range [61]. Surface modification and passivation can directly affect the absorption peaks. Marouzi et al. [86] synthesised the CQD using chia seed and found that the maximum absorption peak of the CQD is near 280 nm due to the transfer of π-π* and the presence of an aromatic functional group. A tiny shoulder peak in the range of 350–370 nm was due to n-π* transfer related to the carbonyl group directing the formation of CQD with the tail of the absorption peak extending to 600 nm. N-Doped CQD formed by the one-step hydrothermal method using glucose and m-phenylenediamine showed four prominent absorption peaks of π-π* transition of C=C at 220 and 270 nm [100], n-π* absorption of C=O of carbonyl and C=N at 290 nm [101], and most importantly, a band gap transition absorption peak at 370 nm [102]. This N-Doped CQD is a direct band gap semiconductor with a band gap of 3.43 eV.

#### 5.1.2. Photoluminescence

**Figure 10:**
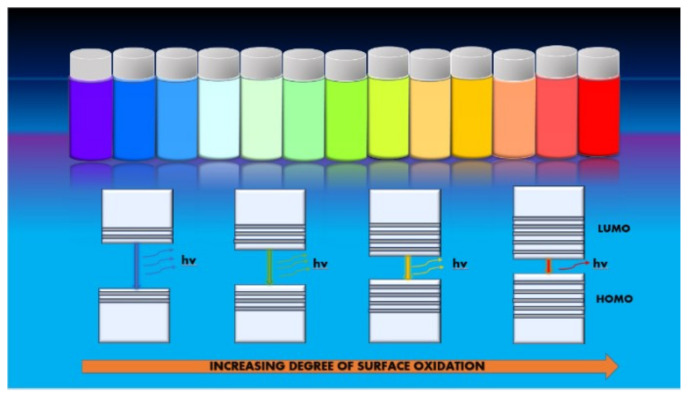
Tunable PL of CQD due to surface oxidation [104].

Photoluminescence is one of the most important and intriguing characterisations for applying quantum dots in multiple fields. The CQDs photoluminescence generally has a feature of excitation-dependent emission wavelength and intensity. The emission peaks of carbon/graphene dots are generally wide with large stock shifts as compared to organic dyes, and there is a distinction between carbon-derived dots and traditional quantum dots based on the PL bandwidth as CQDs have a wider PL bandwidth due to chemical structure and differing PL centres [103]. Ding et al. [104] reported carbon dots with tunable photoluminescence synthesised using the hydrothermal method in one pot and separated via silica column chromatography. The CQDs showed bright and stable luminescence under a single UV wavelength light and high optical uniformity. With similar size distribution, the surface states gradually varied among the different samples, mainly the oxidation degree. Furthermore, a gradual decrease in their band gap along with increasing incorporation of oxygen content on their surface structure resulted in a red shift in the emission peak from 440 nm to 625 nm as their energy bands depended on the surface group and structure instead of particle size (Figure 10). 

**Figure 11:**
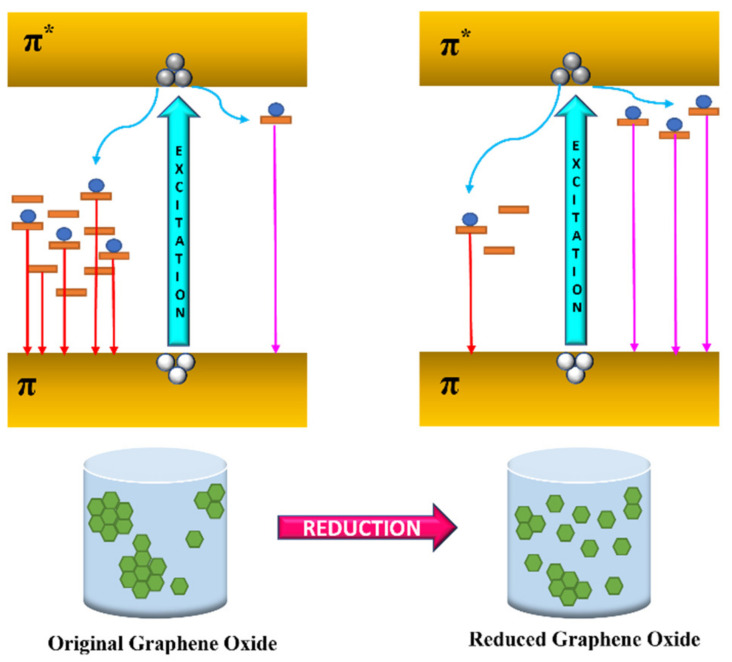
PL mechanism of original and reduced graphene oxide [118].

Sun et al. [42] reported the particle size variation and the photoluminescence emission from the excitation-dependent PL emission. This unique property of CQDs of excitation-dependent PL was widely adopted for multi-colour imaging [105]. The concentration strength of the CQD also plays a vital role in the PL intensity [106]. Many reports have suggested that the excitation-independent emission spectrum might be due to the uniform size and surface properties [47,107]. The principle behind the CQD in light absorption and emission processes relies on an isolated network of π bonding originating from the sp^2^ carbon base. Surface state [108], quantum confinement effect [109], and molecular fluorescence [110] play a crucial role in the excitation-dependent emission behaviour of CQD. Surface passivation using a passivating agent and doping with soft and hard elements can help in tuning the PL of the CQD [111,112]. Reports have suggested that the PL of the carbon dots are pH and solvent dependent as the molecular states are greatly affected by the acidic and basic atmosphere. In contrast, the intensity of PL increased the carbon core-edge state due to the protonation or deprotonation of the various functional groups [113,114]. One report suggested the enhancement of quantum yield and the extension of the emission lifetime by using additives on CQDs with further application in enhanced PCE of PSCs [115]. For graphene-based quantum dots, graphene oxide plays a very important role in GQD’s formation as graphene oxides contain a large fraction of sp^3^ hybridised carbon atoms bounded to oxygen-based functional groups at the edges or on the basal plane, making it an insulator. The various fluorescent properties in such structure alignment are determined by the π state of the sp^2^ sites in which radiative recombination of electron-hole pair can give rise to fluorescence. Reduction of graphene oxide can be achieved via various methods, such as thermal annealing in an inert atmosphere [116], chemical treatment, and photothermal reduction [117]. Gan et al. [118] reported normal graphene oxide solution and its reduction using steady-state Xe lamp irradiation at different exposure times. The PL mechanisms of original graphene oxide and reduced graphene oxide are reported in Figure 11.

The PL mechanism in GQDs can be greatly affected by numerous factors, including pH [119], solvent [113], different surface groups [120], doping with heteroatom [121] and size. Some of the possible phenomena for the PL mechanism in GQD include the quantum confinement effect [122], edge states [67], and surface states [123]. Lai et al. [124] reported excitation and concentration-dependent multicolour photoluminescence in GQDs. GQDs with amino, carboxyl, and ammonium carboxylate groups were synthesised, and it was found that varying the excitation wavelength or concentration of GQDs resulted in distinct luminescent colour. Various characterisation studies summarised that the graphene basal plane and various functional groups exhibited n_N 2P_^_^σ*, π-π*, n_O 2p_^_^π*(-COOH), n_O 2p_^_^π*(-COO-), and n_N 2p_^_^π* electronic transitions, which acted as a multi-fluorescent centre resulting in excitation-dependent multicolour photoluminescence.

#### 5.1.3. FTIR

**Figure 12:**
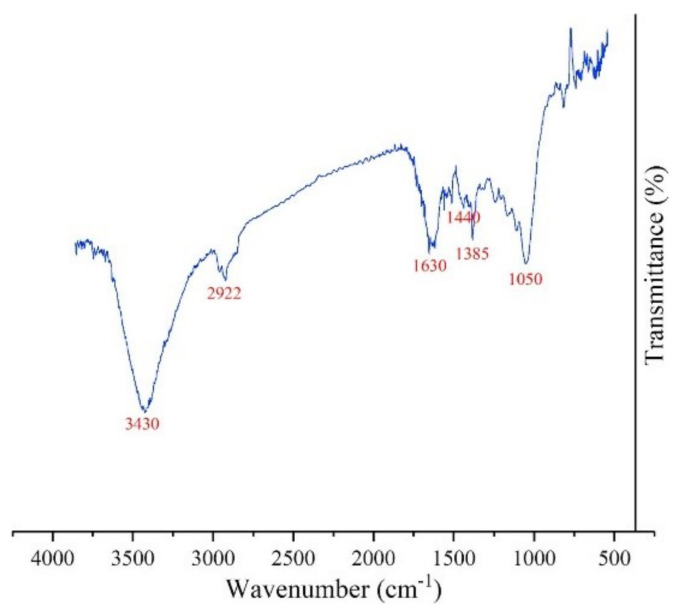
FTIR of carbon dots. Reproduced with permission from [125].

Fourier transform infrared spectroscopy (FTIR) helps analyse the functional group in the formed nanoparticle. CQDs are generally comprised of oxygen, carbon, and hydrogen. Traces of the carboxyl group or carboxylic acid, hydroxyl group, and epoxy group are abundant on the surface of the carbon dots due to the formation of CQD by partial oxidation of the carbon precursors. The FTIR spectrum generally shows prominent peaks around 3378 cm^−1^, 1608 cm^−1^, 1130 cm^−1^, 1710 cm^−1^, and 2850 cm^−1^ corresponding to O-H group stretching vibration, C=O stretching, C-O stretching, C=C in the aromatic ring, and C-H bending, respectively (Figure 12) [125].

### 5.2. Applications

#### 5.2.1. Light Emitting Diode

**Figure 13:**
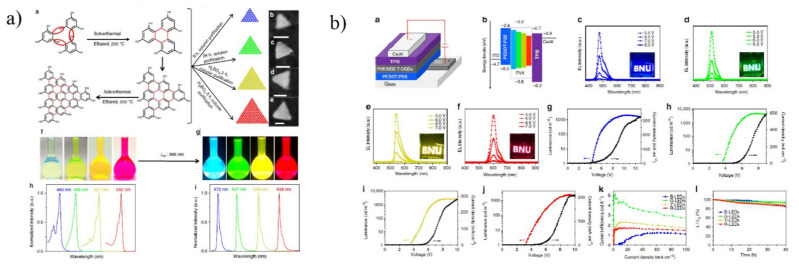
(**a**) Synthesis of CQDs of different colours, fluorescence images under UV light, normalised UV-vis absorption and PL spectra. (**b**) LED structure and various electrical characteristics of the different CQDs. Reproduced with permission from [128].

For optoelectronic applications, CQD/GQDs are a promising candidate for the white light emitting diode (WLED), photovoltaic devices, detectors, and lasers due to features such as broad emission, enhanced thermal stability, tunable band gap, long hot-electron lifetime, and high electron mobility. CQDs generally consist of carbon as a parent source functionalised by different groups present at the edges exhibiting high solubility and reactive sites for various phenomena such as surface passivation and functionalisation [31]. WLEDs are considered the future of solid-state lighting due to their high efficiency, luminance, and energy efficiency with a broad spectrum. The broad emission feature of CQDs due to strong electron-phonon coupling and high dispersive particles as compared to the traditional semiconductor quantum dots (QDs) have attracted intense research for their application in the light-emitting diode field. In the early days, WLEDs were formed using yellow carbon dots on GaN-based blue LED chips [126] but faced drawbacks such as excess blue light emission from the chip and low CRI [127]. Due to various issues such as phase separation, colour fading, sophisticated device design, and limited QY (%), research across the globe has been intensified to solve these issues for promising WLED properties and applications. Yuan et al. [128] reported multicoloured narrow bandwidth emission from triangular carbon dots with a maximum QY(%) of 72%. The high colour purity, narrow bandwidth, and multicoloured CQD were synthesised using solvothermal treatment of three-fold symmetric phloroglucinol as a reagent at 200 °C with different reaction times (Figure 13a). This was combined with a tri-molecular reaction route designed for six-membered ring cyclization propagating to a narrow bandwidth emission labelled as NBE-T-CQD which displayed high colour purity with a maximum luminance of 4762 cd m^−2^ and current efficiency of 5.11 cd A^−1^ (Figure 13b). The size of NBE-T-CQD was controlled using concentrated H_2_SO_4_ in ethanol solution. Due to the quantum confinement effect, with an increase in size from 1.9 nm to 3.9 nm, bright multicoloured emission ranging from blue to red colour was observed with tunability under daylight excitation. The absorption peak was centred at 460 nm for blue, 498 nm for green, 521 nm for yellow, and 582 for red, whereas the emission peak was found to be centred at 472 nm for blue, 507 nm for green, 538 nm for yellow, and 598 nm for red CQDs with an FWHM of around 30 nm. 

**Figure 14:**
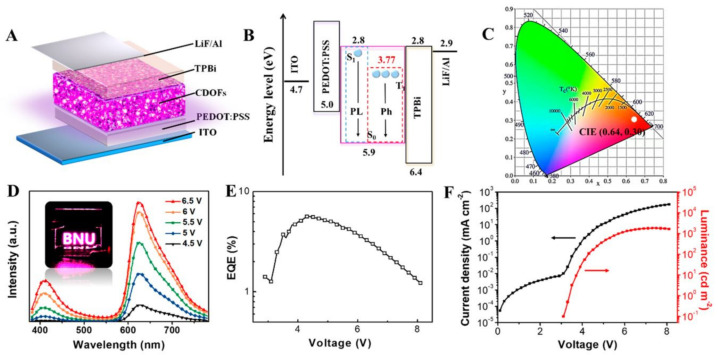
(**A**)LED device setup, (**B**) energy level diagram, (**C**) CIE color coordinates of the carbon dot-based LEDs, (**D**) EL spectra at varied biasing voltage, (**E**) EQE-Voltage relation of the LED and (**F**) Current density-voltage-luminance relation of LEDs. Reproduced with permission from [129].

The bandgap energies decreased from 2.63 to 2.07 eV with excitonic emission peak shifting from 472 nm to 598 nm and size increasing from 1.9 nm to 3.9 nm. For LED, a simple structure was fabricated with NBE-T-CQDs blended with PVK (due to its excellent hole-transporting properties) as the active emission layer. The QY (%) of the blue, green, yellow and red-based NBE-T-CQD with PVK was found to be around 56, 62, 48, and 42%. The final LED structure demonstrated high stability. Shi et al. [129] reported a red phosphorescent carbon quantum dot organic framework (CDOF) with a QY (%) value up to 42.3% and highly efficient red phosphorescent electroluminescent LED with a maximum luminance of 1818 cd m^−2^ and EQE of 5.6% was reported. The author synthesised CDOF by solvothermal treatment 1,3,5-benzenetricarboxylic acid, guanidinium dihydrogen phosphate, and 3,4,9,10-perylenetetracarboxylic dianhydride at 200 °C followed by purification via washing with water, dialysis, and silica gel chromatography. Concentrated H_2_SO_4_ was added to the DMF solution to control the structural morphology of CDOFs. CQDs with an average size of 1.84 nm was observed. For the powder and film state of CDOF in DMF, the absorption spectra showed a major peak centred at 380 nm in the solution state, 395 nm in powder form, and 410 nm for film structure, whereas the PL spectra showed two distinctive emission peaks in which one centred at 625 nm indicating the red emission. The LED devices were formed using ITO glass substrate as an anode, PEDOT:PSS as the hole injection layer, an active CDOF emission layer, TPBi as the electron transport layer, and LiF/Al as the cathode with HUMO and LUMO of the active layer CDOF were at 5.8 and 2.8 eV. The EL spectra of the CDOF-based LED displayed a prominent peak at 425 and 627 nm with a higher intensity of 627 nm, causing the LED to generate red luminescence and showing an apparent voltage-independent emission indicating high colour stability. The maximum current density reached up to 4 cd A^−1^ (Figure 14).

**Figure 15:**
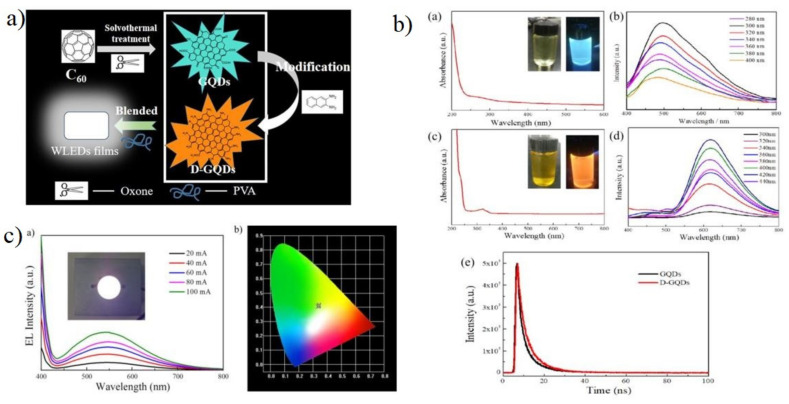
(**a**) Synthesis process of GQD and D-GQD-based composite film. (**b**) Optical characterisation of GQDs and D-GQDs. (**c**) EL spectra of white LED and CIE coordinate of the LED under different driving currents. Reproduced with permission from [133].

Wang et al. [130] reported ethanol and sulfuric acid as a precursor for carbon and carbonising agent, respectively, to synthesise white-emitting carbon dots. Various characterisations showed that the synthesis of white-emitting carbon dots were composed of blue, yellow, and cyan-emitting carbon dots with the same graphitic structure and different surface state due to oxidation and carbonisation, which is responsible for the red shift in the quantum dots emission. Ethanol and sulfuric acids are widely used as a solvent and carbonising agent for the synthesis of high QY (%) of carbon dots [131]. Characterisations further showed that the carbon dots have various polymer structures similar to those found in carbon dots formed via other solvothermal methods. The absorption spectra of different carbon dots showed a peak at 270–300 nm and 300–450 nm. This transition is due to the carbonisation of the C=C bond as an effect of H_2_SO_4_. The time-resolved PL decay shows a double exponential lifetime, and the average lifetime increased from 3.04 ns for blue carbon dots to 7.27 ns for yellow carbon dots. The XPS results showed a gradual increase in C and C=O content with an increasing red shift in carbon dots’ emission wavelength. For the fabrication of LED chips, the carbon dots were dropped into epoxy resin and well stirred, followed by drying in an oven for 20 min. Furthermore, UV chips were amalgamated with the formed carbon dot/epoxy composites. Wang et al. [132] synthesized WLED by mixing various kinds of CQDs prepared through a one-step acid reagent by heating a mixture of oPD and appropriate acid reagents such as folic acid, boric acid, acetic acid, tartaric acid, and terephthalic acid in an ethanol solution. Based on the variation of acid reagents, six CQDs with blue (b-CQDs), cyan (c-CQDs), yellow-green (yg-CQDs), orange (o-CQDs), red (r-CQDs) and white (w-CQDs) fluorescence were synthesised. The average size of the CQDs ranged between 1.71 nm to 2.42 nm. Most of the solution prepared emitted blue and green fluorescence with dim intensity. Due to the presence of strong acids, excessive carbonisation and defect formation occur during the reaction. The surface of CQDs is composed of the electron-withdrawing group, which helped in enhancing the photoluminescence wavelength red shift and an increment in the particle size. The absorption and emission spectra of various CQDs formed exhibited a strong absorption and the emission band at b-CQDs (377 nm, 450 nm), c-CQDs (428 nm, 490 nm), yg-CQDs (454 nm, 540 nm), o-CQDs (571 nm, 600 nm), and r-CQDs (628 nm, 665 nm). The band gap energy of the different CQDs gradually decreased from 2.76 to 1.88 eV with an increase in particle size. The HOMO-LUMO level also displayed a similar decreasing trend from 5.22 to 3.83 eV and from 2.46 eV to 1.95 eV. Using the time-resolved PL, w-CQDs exhibited a mono-exponential fluorescence lifetime of 3.07 ns. The formed CQDs emitted bright and stable full colour ranging from blue to red light and even white light. For the WLED, the pure CQDs and those with various ratios were implemented to form full-colour PMMA nanocomposite film. Except for the white light emission film, two white luminous films were also achieved by varying CQD ratios. The WLEDs were synthesised using CQDs in silica gel in different concentrations and mixtures. WLEDs can be easily formed by anchoring the w-CQDs phosphor onto the UV-based blue chips. The w-CQDs maintain excellent photostability and thermal stability with a broad PL ranging from 360 nm to 800 nm. Three different kinds of WLED were formed: warm WLED using o-CQDs and blue LED chips, whereas standard and cool WLEDS were formed using w-CWDs mixed with CQDs with b-CQD/yg-CQD/r-CQDs = 1:2:1 in ratio and UV-LED chips. Chen et al. [133] reported the potential application of graphene quantum dots for commercial white LEDs. They proposed a technique to synthesise uniform blue-green emissive GQDs and orange-emitting GQDs (D-GQDs) by using fullerene (C_60_) as the carbon source under acid-free conditions (Figure 15).

The quantum yield was as high as 52.4%, with an emission peak of 617 nm. The persulfate-assisted one-step redox method was adopted to open C_60_, as a result of which, GQDs with uniform size were obtained and further modified using 2,3-diaminonaphthalene (DAN) to tune the emission wavelength and enhance the quantum yield. For the application of white-light LED, fluorescent films were fabricated via mixing the different types of GQDs formed with ethylene glycol. Later, they were combined with UV chips for the final structure. The TEM analysis shows that the GQDs and D-GQDs were homogenous spherical dots in shape with no aggregation, with an average size of 3.21 nm for GQDs and 3.5 nm for D-GQDs. Absorption spectra showed absorption in the range of 250 to 320 nm for the GQDs, whereas it was between 271 and 323 nm for the D-GQDs. From the bandgap calculation, the bandgap was 3.81 eV and 3.44 eV for GQDs and D-GQDs, respectively. Furthermore, the GQDs showed excitation-independent photoluminescence. The light emission colour from the GQDs and D-GQDs were cyan and orange. Thus, white light emissions can be formulated by mixing the quantum dots in a specific ratio. When done so at an excitation wavelength of 384 nm, the PL peaks were located at 480 nm and 580 nm. The colour coordinates were also dependent on the ratio between GQD and D-GQDs. For the WLED application, the GQDs/D-GQDs/PVA films, along with LED chips (centre emission wavelength of 380 nm), were amalgamated. The EL spectra and CIE coordinates were recorded for different driving currents. Additionally, the colour shift was calculated to check the colour stability of the LED device. By changing the range of current from 20 mA to 100 mA, the colour shift was 9 × 10^−3^, exhibiting good optical stability. The CIE coordinate for the current value of 80 mA was (0.34, 0.40), located in the white light region. The colour temperature was found to be low as 5290 K with a CRI up to 77.3 for the combination of the luminescent film with UV LED at 80 mA. Single-component white emission carbon quantum dots (SCWE-CQDs) have drawn an immense amount of attention in the past few years. Rao et al. [134] reported fast and efficient synthesis of SCWE-CQDs with high photoluminescence by controlling the dilution ratios of N,N-dimethylformamide in pristine red CQD (RCQD) solution.

The RCQDs were formed using 5.2 g of AC in 50 mL of DMF solution, which was then transferred to a Teflon-lined stainless-steel autoclave and heated at 200 °C for 6 hrs resulting in a red solution. Furthermore, N-(b-aminoethyl)-g-aminopropyl methyl dimethoxy silane was adopted as a solvent and protective material to inhibit fluorescence self-quenching and the preparation of SCWE-CQDs. They achieved a PLQY of 53% by optimising the synthesis route. Due to the hydrogen bond effect and size effect, a blue shift in RCQDs was observed with a large-spectral shift. For the WLED device formation, different CA contents were added to the formed SCWE-CQD solution and stirred well. The homogenous solution was then drop-casted on a 395 nm UV-LED chip and heated at 130 °C for 2 hrs for solidification. The TEM analysis shows that the narrow size distribution of RCQDs and SCWE-CQDs were 3.47 nm and 2.65 nm in diameter, respectively, with a sign of cluster formation in the former case due to aggregation. In the absorption spectra, with an increase in DMF content, a gradual blue shift was observed in the case of RCQDs with a distinct absorption band at 621 nm for no DMF addition, which further shifted to two distinct peaks at 536 nm and 593 nm as the dilution ratio increased from 50 to 100 times. A further increase in the dilution resulted in an absorption peak in the UV region at 318 nm. The PL emission spectra of the RCQDs were centred at 675 nm for no DMF dilution, which was excitation-independent. With an increase in dilution up to 5 times, the PL spectra showed two sharp peaks at 540 nm and 655 nm, with a higher intensity in the latter. With an increase in the dilution rate of DMF, the PL spectra showed multiple peaks and were blue-shifted. For a 100-times dilution, the colour of the solution turned pure white, resulting in the formation of SCWE-CQDs. This result demonstrates that the white light of the SCWE-CQDs was composed of a mixture of colours. Finally, the performance of the WLEDs was affected by the concentration of cellulose and SCWE-CQDs. Therefore, varying the concentration of one while keeping the other was performed and by further optimising the content of both, WLEDs with high CRI (up to 89) and reliability were achieved. Yin et al. [135] reported a tunable colour temperature (3196–10,870 k) white light-emitting diode using a thin polymer film with high optical transparency from yellow-green emitting GQDs. The GQDs showed a slight red shift in the emission spectra when the excitation wavelength was varied. In the absorption spectra, there was a strong peak at 324 nm and a weak absorption peak at 407 nm, corresponding to chlorine doping. For the excitation spectra, the GQDs showed peaks at 330 nm and 409 nm. Spectral tuning was clearly obtained by varying the concentration and thickness of the GQD film. For the stability of formed quantum dots, the GQDs were introduced to a UV-curable adhesive matrix. To achieve a transparent GQD/UV glue mixture system, 200 µL of GQD toluene solution at a concentration of 24 mg mL^−1^ and 2 g of UV was centrifuged and well stirred with further heating at 50 °C for the removal of toluene. Finally, the GQD/UV glue composite was deposited on the blue LED resulting in a white LED as the final device.

In recent years, carbon dots—due to carbons’ high stability, low cost, and eco-friendly nature—have found practical applications in the field of OLED technology [136]. The carbon dots (CDs) are widely used as emitter, both as a neat layer and as a guest, sometimes as a charge regulating interlayers. Researchers have also used CDs as remote emitters, providing traditional blue LED with a CD-based colour-converting layer during its application in OLED, where the blue emission was widely tuned by varying the film thickness of the filter or by changing the doping concentration [104,137,138]. Wang et al. [139] fabricated white OLEDs from a carbon dot film that was fabricated with a high PLQY of up to 60% by using carbon dots as an emitting layer by thermal carbonisation of CA in octadecene with HDA adopted as a passivating agent. The current efficiency of the white OLED was 0.022 cd/A, and the EQE was 0.083% at a current density value of 5 miliAmp cm^−2^. Zhang et al. [140] reported a multi-coloured EL-based OLED in which carbon dots were used as an emissive layer between an organic HTL and organic or inorganic ETL with a structural design of ITO/PEDOT:PSS/polyTPD/CDs/TPBI or ZnO/LiF/Al. The emitted colour was tuned by changing the thickness of LiF or by substituting LiF and TPBi with ZnO.

In the last five years, microLEDs have also attracted researchers due to their promising properties in the field of brightness, lifetime, efficiency, and higher resolution as compared to present sources of display technology. Indium-doped GaNs are widely used materials for violet and blue LEDs because of their higher efficiency, longer lifetime, self-emissivity, and reliability under unfavourable environmental conditions. Jiang et al. [141] reported an InGaN-based blue LED with an EQE value of over 80%. Recently, phosphor-converted LEDs and full-colour microLED devices have taken over research by employing red-green-blue (RGB) microdisplays. Colloidal quantum dots are emerging as an emissive material that can replace organic or phosphor-based LEDs. Due to limitations in current LCD and OLED technologies based on backlight necessity and lower response time, respectively, micro-LEDs can provide high contrast, wide colour gamut, high response time, and wide viewing angle [142,143]. Among the different types, one type of micro-LED works on RGB colours by distributing colour-converting patterns on monochromatic UV-based micro-LED arrays. Heavy metal-based quantum dots are not adopted due to their harmful effects on the environment. Liu et al. [144] reported red-emitting carbon dots with an emission at 635 nm with a QY (%) value of 30%. Absorbance was observed in the UV range with a stokes shift of 230 nm. The as-prepared carbon dots were soluble in organic solvents and did not bleach under long-term excitation. Furthermore, the carbon dots were incorporated in a UV-curable polymer. For their application as red emitters, the carbon dot-embedded polymer pattern was cured using fast photocuring technology. Dai et al. [145] reported a phosphor-converted white light-emitting diode (pc-WLED) using rare-earth-free phosphor GQDs and copper-cysteamine (Cu-Cy) via a microwave-assisted wet-chemical method in which graphite was used as carbon source. Under an excitation wavelength of 365 nm, warm white light was attained by mixing the two self-activated RE-free phosphors in a specific weight ratio of 1:1.7. The LED device exhibited an EQE of 0.31% with R= 87.9, T = 4436 K, and CIE(x,y) = (0.341,0.327).

#### 5.2.2. Energy Storage Application

In today’s world, there is an exponential increase in energy demand and progress in the field of sustainable and green energy resources for the future as fossil fuels are running short, and their environmental effects raise a lot of concern and are becoming a severe challenge to tackle in the near future. Recently, electrochemical energy storage devices have gained a lot of attraction due to their exciting and promising features, such as low-cost fabrication, high power density, high energy, and economical and fast charging–discharging capabilities [146]. Looking from a power density and energy point of view, batteries and capacitors are the commonly adopted energy storage devices, with batteries having high energy density and low power density, whereas capacitors have high power density and low energy density [147]. In recent years, to address this issue, supercapacitors have been developed, which can have high energy and power densities, exceptional charge storage and cycling stability, and an increase in the development of flexible and wearable electronic devices; flexible energy storage devices are the main attraction for most [148]. To date, various studies have been conducted on the simplification of design and construction of nanomaterials based on GQDs for their use as electrodes. Along with the different semiconductor-based quantum dots, oxides based on metal oxides and composite systems with carbonaceous materials have been adopted for electrode preparation for capacitors, supercapacitors, and batteries so far [149,150,151]. Not only this, inorganic materials such as nanowires, perovskites, and metal-organic frameworks (MOFs) have also been a choice for electrode material for energy storage devices [152,153]. To increase the overall performance of the devices, carbon and graphene-based quantum dots have been used for the fabrication of economical, high-capacity, sustainable, and long life cycle electrodes for their application in energy harvesting.

#### 5.2.3. Supercapacitors

A charge storage device that can store energy in two ways—electrostatically (electrical double layer capacitors (EDLC)) or electrochemically (pseudocapacitors)—is defined as a supercapacitor, which basically comprises two electrodes immersed in an electrolyte that may be separated using a separator, which plays a crucial role in the supercapacitor’s performance [154,155,156,157]. EDLCs are conventional capacitors in which the capacitance develops from electrostatic charge accumulation between the ions at the interface, giving infinite time charge/discharge; they are stable from a life cycle stability point of view, but due to restricted specific surface area and compatible electrodes, EDLCs faces lower energy densities compared to pseudocapacitors [158]. Pseudocapacitors work on the principle of a reversible faradaic redox reaction arising from electroactive phases at the interface and suffer from cycling stability [159]. In the mid-19th century, the first electrochemical-based capacitor with low voltage and high capacitance was reported to be made up of porous carbon-based electrodes [160]. Using various sources, e.g., inorganic materials or carbon-based materials, has some limitations, such as poor life cycle and low capacitance. For sustainable and green energy storage devices, carbonaceous-based materials, such as carbon and graphene-based quantum dots, have attractive properties, including tunable electrical and optical properties, highly bio-compatible, stable, large surface-to-volume ratio, high conductivity, and flexibility. They have applications in biosensors and bioimaging and can meet the demand for ideal electrodes (i.e., high energy and power densities, large capacitance, and long life cycle). 

**Figure 16:**
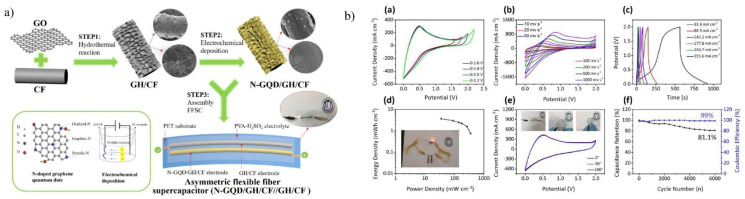
(**a**) Fabrication steps of the GH/CF negative electrode and N-GQD/GH/CF positive electrode with design of an asymmetric flexible fibre supercapacitor. (**b**) Two-electrode electrochemical performance of asymmetric N-GQD/GH/CF//GH/CF FFSC in H_2_SO_4_/PVA electrolyte. Reproduced with permission from [166].

With the innovation and development of diverse wearable and flexible electronic devices based on micro and nanotechnology in recent years, the demand for flexible energy storage has also increased exponentially. For flexible electronics media, flexible supercapacitors can attain high mechanical and electrochemical performance properties. The flexible electrodes are basically fabricated based on three different structural designs: one-dimensional fibre structure, two-dimensional plane structure, and three-dimensional laminated structure for different applications. Flexible supercapacitors based on graphene—due to its high specific surface area, high conductivity, and mechanical properties—have shown some exciting features such as fast charging and discharging, long life cycle, highly flexible, and bendability with high power density [161]. Electrode materials being an important part of supercapacitors, can be of different compositions based on carbon materials, conductive polymers, and transition metal oxides. For EDLC, carbon materials are preferred, but their specific capacitance is low, resulting in low power density, whereas, for pseudocapacitors, transition metal oxides and conductive polymers are preferred and have advantages such as high specific capacitance and large voltage range but show poor cycle stability at high current ranges [162,163,164]. Davies et al. [165] reported a flexible uniform graphene-polypyrrole composite film for supercapacitor electrodes using the pulsed electro-polymerisation method. The flexible supercapacitor exhibited high energy density (33 Wh kg^−1^) and power density (1184 W kg^−1^) at a scan rate of 0.01 Vs^−1^. Li et al. [166] fabricated all-carbon fibre electrode (N-GQD/GH/CF) structures in which graphene hydrogel (GH) was grown on carbon fibre (CF), forming a 3D interconnected porous (GH/CF) network (Figure 16). Additionally, a nitrogen-doped graphene quantum dot with high pseudocapacitive activity was electrodeposited on the formed network. The so-formed structure resulted in enhanced electrochemical performance; N-GQD/GH/CF was used as the positive electrode and GH/CF as the negative electrode. Recently, immense research has been going on in the field of novel capacitors, including all-solid-state capacitors and micro-supercapacitors, which can be derived from GQD-based materials, as GQD-based supercapacitors can have energy densities close to batteries.

Luo et al. [167] suggested a fabrication technique of graphene quantum dots based on three-dimensional graphene (3DG) using a hydrothermal method. The synthesised composite setup of GQDs/3DG as an electrode for supercapacitors displayed high electrochemical stability with a capacitance of around 93% after 10,000 charge-discharge cycles. Due to the bright potential in the field of micro- and nano-range energy storage devices, micro-supercapacitors are drawing attention. Still, their low energy density limits their practical application to a great extent. Shen et al. [168] fabricated all-solid-state asymmetric micro-supercapacitor using graphene quantum dots and MnO_2_ nanosheets as negative and positive electrodes, respectively, with different ionic liquids as the solid electrolytes, whereas Liu et al. [169] reported GQD//GQDs symmetric micro-supercapacitors using a simple electro-deposition technique. The as-formed GQD micro-supercapacitors showed an admirable rate capability up to 1000 V s^−1^, great power response with a short relaxation time constant of 103.6 µs in aqueous electrolyte, and 53.8 µs in ionic liquid electrolyte with exceptional cycle stability. Another setup with a GQD//MnO_2_ asymmetric supercapacitor was fabricated with MnO_2_ nanoneedles as the positive electrode and GQD as the negative electrode in an aqueous electrolyte. Compared to the GQD//GQD supercapacitor, it showed a double-fold increase in specific capacitance and energy density. When carbon quantum dots are doped with elements such as nitrogen or sulphur, they typically become and act like pseudo-capacitors. Li et al. [170] fabricated highly pseudocapacitive material using N-doped GQD, with carbonised ZIF-8 as a self-sacrificing template and CNT as a conductive network for the supercapacitor. The formed supercapacitor had a high specific capacitance of 540 F g^−1^ at 0.5 A g^−1^ in 1 M H_2_SO_4_ electrolyte and showed excellent cycle stability after 8000 continuous cycles with a high energy density of 18.75 W h kg^−1^ and power density of 108.7 W kg^−1^. High-surface area carbons are generally preferred for super-capacitors, but due to their amorphous microporous structure, they exhibit limited capacitive and rate performance because of low conductivity and electrochemical kinetics. Qing et al. [171] reported enhancement in the electrochemical performance of the activated carbon by the use of graphene quantum dots. As a result of which, activated carbon embedded with graphene quantum dots with a specific surface area of 2829 m^2^ g^−1^ attained high electric double layer capacitance of 388 F g^−1^ at 1 A g^−1^ with a significant rate performance of 60% capacitance retention at 100 A g^−1^ in a two-electrode system. The remarkable performance of the system was attributed to the increased charge transfer and ion-migration kinetics of activated carbon due to the formation of the overall conductive network. Yin et al. [172] fabricated a hybrid structure NiO@Co_3_O_4_@GQD sphere using a one-step solvothermal method for application in supercapacitors. The introduction of carbonyl-functionalised GQDs helps in the production of more active sites, improvising the electronic conductivity, and amplifying the ion accessibility. Using Co-Ni-based MOF as a precursor for the synthesis of NiO@Co_3_O_4_ with a multi-layered hollow structure, GQD was further introduced into the system as a cathode for use in supercapacitors. NiO@Co_3_O_4_@GQD displayed a specific capacitance of 1361 F g^−1^ at 1 A g^−1^ and maintained a capacitance retention rate of 76.4% after 3000 cycles. Additionally, all-solid-state ASC (NiO@Co_3_O_4_@GQD//AC) demonstrated a maximum energy density of 38.44 Wh kg^−1^ with a high cycling capability (retention rate of 84.3% after 10,000 cycles).

Zhang et al. [173] developed a strategy for the fabrication of a hierarchical porous carbon nanosheet (HPCN) for supercapacitors. Using bituminous coal as a precursor, coal-based GQDs were prepared by simple liquid phase oxidation. Due to attractive features such as small size, enhanced edge structure, ample functionals group with good chemical reactivity, and high flexibility, they showed potential applications in the field of advanced energy storage materials. The HPCN displayed an interconnected graphene-like network with a surface area of 1332 m^2^ g^−1^ with pore distribution, excellent conductivity, diverse active sites, and various channels for ion migration. The synthesised device exhibited a specific capacitance of 230 F g^−1^ at a current density of 1 A g^−1^ and capacitance retention of 74% at 100 Ag^−1^ with no capacity reduction after cycling at 10 A g^−1^ 10,000 times, ensuring high endurability. Islam et al. [174] reported the chemical synthesis of graphene quantum dots on carbon fibre (CF) in situ using covalent ester linkages at 90 °C. The formed flexible fabric supercapacitor using grafted material exhibited enhanced electrochemical capacitance due to features such as active surface sites in GQDs compared to carbon fibre and defect creation resulting in GQD recombination inhibition. The retention rate was almost 97% after 5000 cycles at 2 A g^−1^ current density, demonstrating its stability potential for use in an advanced flexible electronic device. High-rate capability at high mass loading is one of the important parameters for advanced supercapacitors in the near future. Tian et al. [175] studied a micelle-induced assembly technique for the development of conductive porous carbon through graphene quantum dots with GQDs as a precursor and block copolymer F127 as soft templates. The hybridisation state (sp^2^) of GQDs helped in promoting the electrical conductivity to multiple folds, as compared to activated carbon. The formed interconnected mesoporous structure exhibited a high specific surface area of 1323 m^2^ g^−1^ and remarkable electric conductivity of 73 S m^−1^ and assisted vigorous ion transport kinetics at high mass packing. As an electrode in a supercapacitor, it displayed high capacitances of 315 and 170 F g^−1^ at 1 and 100 A g^−1^, respectively. The symmetric supercapacitors showed maximum energy densities of 6.45 Wh kg^−1^ and 9.21 Wh kg^−1^ at mass loading of 20 mg cm^−2^ and 2 mg cm^−2^, respectively. This experimental data discloses the advantage of the GQD-puzzled porous carbon structure for practical applications in the near future. Li et al. [176] fabricated an N-GQD/c-MOF-5 composite via the electrodeposition method using highly N-doped GQDs on porous carbon obtained from carbonised MOF-5 as an electrode material. This composite electrode showed features such as high capacitance, high-rate capability, high energy and power densities, and a long life cycle. The asymmetric supercapacitor formed using N-GQD/c-MOF-5 as a positive electrode and AC as the negative electrode was found to operate at a wide potential of 1.6 V with an energy density of 14.4 Wh kg^−1^ at a power density of 400.6 W kg^−1^ with a retention of 90.1% after 5000 cycles.

Zhang et al. [177] reported the effect of the presence of acidic oxygen-bearing functional groups such as -COOH and OH on GQD, which could serve as a solution and solid-type electrolyte for various supercapacitors. The GQDs were synthesised using the chemical oxidation of graphitic oxide. Neutralising the acidic functional group using KOH can enhance the ionic conductivity and ion-donating ability of GQDs. These neutralised GQDs strengthen and enhance the supercapacitors’ capacitive performance and rate capability, which can be attributed to fully ionising the weak acidic oxygen-containing functional groups after the neutralisation process. 

#### 5.2.4. Batteries

Batteries are one of the storage devices where most renewable energy harvested is stored. Batteries do not need actual stored electrical energy; rather, they store energy in the form of chemical energy inside electrode active materials through chemical bonds and convert it into electricity via different processes such as the redox reaction or intercalation technique. It is important to develop a supercapacitor battery technology with features such as high efficiency, long-term cycling life, low weight, and long-term stability with high energy and power density [178,179]. The two different processes (redox reaction or intercalation technique) can be found in Table 1. Lithium-ion [180,181,182,183,184] or sodium ion-based batteries [185,186] and their conventional structure can be described as containing two electrodes separated by an electrolyte [187].

Among the different kinds of batteries, lithium-ion batteries have been accepted for daily uses in portable devices for their high storage capacity and energy density, long life cycle, and low cost. Due to their unique morphology, high surface area, and high structural stability and conductivity, carbon nanotubes and graphene play a crucial role in increasing the lithium-ion battery’s capacity. To date, carbonaceous materials have been widely used for fabricating efficient electrodes for Li-ion and Na-ion batteries. Electrode materials are very important for batteries. Graphite has been preferred as anode material because of its economical and structural stability, and quantum dots based on graphene also play an important role due to their large surface area and ease of functionalisation. Additionally, to enhance the performance of the batteries, C-dots have also been preferred for active electrodes. Carbonaceous materials with large interlayer spacing and disordered structures are preferred for the electrode in sodium-ion batteries, which can tackle the problems faced by using conventional electrodes. Thangaraj et al. [188] reported flexible Na-ion batteries with SnO_2_ and NaVO_3_-coated carbon quantum dots as anode and cathode materials. The carbon quantum dots were synthesised via hydrothermal techniques and alkaline peroxide treatment using dead leaves of the samanea saman tree. The quantum yield was found to be 21.3% at an excitation wavelength of 360 nm. CQDs, CQD@ SnO_2_, and CQD@ NaVO_3_ exhibited excitation-dependent wavelengths with crystalline sizes 12.8 nm, 11.9 nm, and 14.4 nm, respectively. Five different separators (ITO/PTE, RP, SIL BH, SIL CH, and CP) performances in Na-ion batteries were examined, and the CV results showed that the silicone-based separator SIL SH gave a higher specific capacitance value (881 F g^−1^) as compared to SIL BH (116 F g^−1^). Studies show that the charge and discharge specific capacities were reduced in RP, SIL BH, and CP at 2 V. SIL SH showed a high initial specific discharge capacity in the 1st and 10th cycles (4246 and 3003 mC g^−1^) at 2 V but showed lower specific discharge capacities in the 25th and 50th cycles (130 and 71 mC g^−1^) compared to CP and SIL BH. Sodium-ion batteries are preferred due to the low sodium cost and abundant resources. However, there are drawbacks, such as low energy density and undesirable cycling capability with slow ion transfer. 

Liu et al. [189] reported Na_3_V_2_(PO_4_)_2_F_3_-based carbon quantum dot microspheres synthesised using the one-step solvothermal technique followed by heat treatment. The NVPF@CQD electrodes displayed high-rate capability with a discharge capacity of 105.1 mAhg^−1^ at 20 C, indicating an 83% retention. When the cycling rate increased to 30 C, the retention rate was reported to be 90.2% after 6000 cycles. Saroja et al. [185] presented the fabrication of graphene quantum dots and heteroatom-doped graphene quantum dots using chemical vapour deposition. With methane as a carbon precursor, the GQDs were prepared using an alloy-based catalyst. Boron and nitrogen-doped graphene quantum dots were synthesised using graphite oxide at low temperatures. For their application in Na- and Li-ion storage, various properties of undoped and doped graphene quantum dots were examined. The B-doped GQDs showed a high specific capacity of 1097 mAh g^−1^ and 310 mAh g^−1^ at a specific current of 50 mA g^−1^ for lithium and sodium ion batteries, respectively. Furthermore, boron-doped GQDs exhibited high volumetric energy densities of 537 AhL^−1^ and 214 Ah L^−1^ with average voltage for lithium and sodium ion batteries of 0.43 V and 0.57 V, respectively. The presence of edge defects on the formed quantum dots helped adsorb metal ions, and additional doping increases the insertion process, which results in a specific capacity.

**Figure 17:**
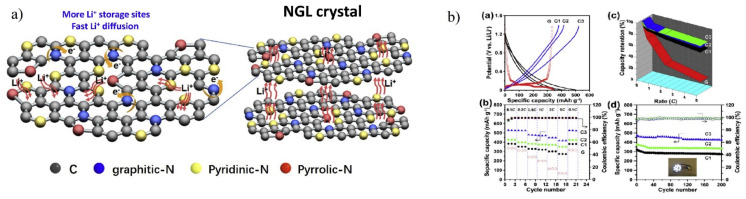
(**a**) Li storage mechanism in 3D NGL crystals. (**b**) (a) Charge-discharge curves of traditional graphite and NGL anodes at 0.1 C, (b) specific capacity and coulombic efficiency as a function of cycle number at different rates, (c) capacity retention as a function of C rate, (d) cyclic stability of different NGL anodes at 1 C. Reproduced with permission from [195].

Due to their high energy density, environmentally friendly nature, and good cycling stability, lithium-ion-based batteries are one of the most useful power sources. Till now, due to self-aggregation, dissolution and rapid increase in the charge transfer resistance during cycling, the cathode material faces issues such as poor rate performance and rapid capacity fading. In contrast, for the anodic materials, electrolyte depletion rate and low coulombic efficiency are the major concerns. To overcome these issues, up to a certain extent, carbon dot-based materials are a promising candidate for cathode/anode materials. Carbon dot-based materials are also used as electrolytes for lithium-ion batteries by modification of the internal structures and surface states of the electrode materials and as additives to electrolytes, improving the overall performance of Li-ion-derived batteries [190]. Chao et al. [186] fabricated a binder-free cathode using the bottom-up approach of a biface VO_2_ array directly on a graphene network for better performance in lithium-ion and sodium-ion batteries. The graphene quantum dots were further coated onto the VO_2_ surface as a surface sensitiser and protection, which helped enhance the electrochemical properties. The unified electrodes supply a Na storage capacity of 306 mAh/g at 100 mA g^−1^, with a capacity of more than 110 mAh g^−1^ after 1500 cycles at 18 A g^−1^. The low specific capacity and depressed rate capability of carbonaceous materials reported in potassium-ion batteries limit the potential of carbon-based materials. Hong et al. [191] investigated the potassium storage properties of carbon materials and carbon as an anodic material for potassium-ion batteries. It was reported that the hollow nanostructured N-doped carbon (p-HNC) was synthesised using carbon quantum dots and CQD micelle formation. The hydrophobicity and lipophilicity helped as a template for hollow structure formation and acted as a pore-forming agent due to the decomposition and contraction of CQDs during pyrolysis for the creation of micro-tunnels. For potassium storage, the (p-HNC) anode exhibits high reversible capacities of 254 mAh g^−1^ at 0.1 A g^−1^ after 100 cycles and 160 mAh g^−1^ at 1.0 A g^−1^ after 800 cycles. Other measurements showed an improved diffusion coefficient of p-HNCs, indicating their superior kinetic property. Prasath et al. [192] synthesised nanostructured bismuth oxide (Bi_2_O_3_) and carbon quantum dots (CQD) via the hydrothermal technique using denatured milk-derived CQDs. The Bi_2_O_3_—CQDs composite was investigated as an anodic material in lithium-ion batteries, and it was found that the composite offers high electrochemical activity exhibiting a discharge capacity as high as 1500 mAh g^−1^ at a 0.2 C rate. The Bi_2_O_3_–CQD composite showed good reversibility and a high specific capacity of 343 C g^−1^ at 0.5 A g^−1^ in 3 M KOH for supercapacitors application measured in a three-electrode system. Furthermore, the asymmetric device fabricated using CQD–Bi_2_O_3_ and reduced graphene oxide delivered a maximum energy density of 88 Wh kg^−1^ at a power density of 2799 W kg^−1^, while the power density reached a maxima of 8400 W kg^−1^ at an energy density of 32 Wh kg^−1^ in the potential window range of 0–1.4 V. Graphene quantum dots (GQDs) have been preferred as a composite or coating material in storage devices due to their promising properties such as inducing the finite band gap in the material, which changes the electronic conductivity due to the quantum confinement effect. This effect influences the lithium diffusivity resulting in the battery’s electrochemical performance and long-term electrochemical cycling [193]. Furthermore, the addition of GQDs accelerates the large electron transfer and the electrolyte transport to the respective electrodes resulting in improvising the electrochemical performance of the Li-ion batteries. Using GQD as an electrode coating material results in the production of a large surface area for ion transfer between the electrolyte and active materials, further attaining ultra-fast energy storage and release [194]. Gu et al. [195] reported nitrogen-doped graphite (NGL) electrode materials via the solid-phase microwave-assisted method followed by thermal pyrolysis of N-functionalised graphene quantum dots and was examined as the anode for Li-ion batteries (Figure 17).

The as-formed NGL anode displayed a reversible capacity of 530 mAh g^−1^ at 0.1 C, superior rate capability at high C rate operation (420 mAh g^−1^ at 5 C), and exceptional initial coulombic efficiency (>95.7%) with remarkable cycling stability along with very high efficiency (99.1%) during the entire cycling. The NGL anode material helped improve lithium-ion mobility and reversible Li^+^ storage during cycling. The specific energy of the anode reached 840 Wh kg^−1^ at the power density of 4200 W kg^−1^. The D_Li_ value of the NGL anode was found to be 1.69 × 10^−9^ cm^2^ s^−1^ (15–26 times higher than the graphite anodes).

**Figure 18:**
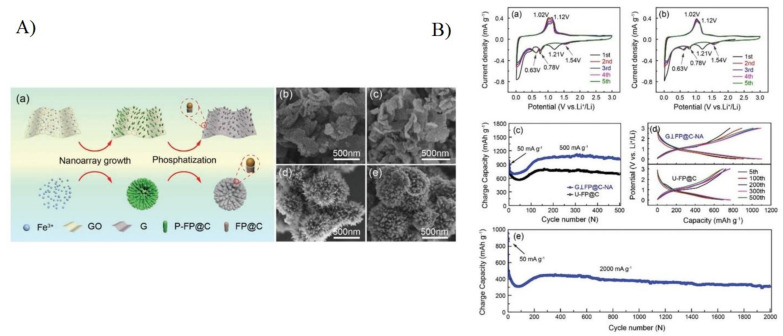
(**A**) (a) Formation process of the G⊥FP@C-NA and U-FP@C with SEM images. (b) P-G⊥FP@C-NA, (c) G⊥FP@C-NA, (d) P-U-FP@C, and (e) U-FP@C. (**B**) CV curves of the initial five cycles at 0.1 mV s^−1^ and long-term cycling stability. Reproduced with permission from [196].

Hou et al. [196] reported a unique nanocomposite named G⊥FP@C-NA, in which a carbon-coated FeP nanorod array was vertically grown on a 2D conductive reduced graphene oxide network using a scalable strategy (Figure 18). The composite displayed enhanced conductivity, structural stability, and pseudocapacitance-boosted ultrafast electrochemical kinetics for Li storage.

G⊥FP@C-NA revealed a high capacity (1106 mAh g^−1^ at a current density of 50 mA g^−1^), exceptional long-term cycling stability (1009 mA h g^−1^ at 500 mA g^−1^ after 500 cycles and 310 mA h g^−1^ at 2000 mA g^−1^ after 2000 cycles), and excellent rate capability (565 mA h g^−1^ at 5000 mA g^−1^) as an anode material for Lithium-ion batteries. The array structure designed for this composite can also be further extended for its use in various energy storage systems, such as sodium/potassium ion-based batteries and supercapacitors with high power and high energy densities. Electrodes in powdery form require a binder and conductive additives, which results in the cells’ low and unsatisfactory energy storage performance.

Balogun et al. [197] recently fabricated VO_2_ interwoven nanowire surfaces engineered with carbon quantum dots (CQD) on 3D flexible carbon cloth via the hydrothermal technique and showed its potential as a cathodic material for lithium and sodium-ion batteries. Being flexible for surface engineering, the formed carbon quantum dots also played an important role in the diffusion of electrons and enhanced the storage performance of Li- and Na-ion batteries. The flexible carbon cloth demonstrated bottom-up support for binder and additive-free growth of interwoven nanowires. The as-synthesised C-VOCQD electrodes exhibited a high capacity of 173 mAh g^−1^ at 60 C. They maintained more than 100% of their initial capacity after 500 cycles for Li storage and 80% after 200 cycles for Na storage. The energy and power densities of the C-VOCQDs for Na storage extended to 264 Wh kg^−1^ and 41,333 W kg^−1^. The results showed a promising approach for developing economical sodium-ion batteries for next-generation post-lithium-ion batteries. Silicon, a favoured anode material for high-capacity lithium-ion batteries, has the disadvantage of large volume variations during the charge–discharge process, resulting in a fast capacity loss on cycling and electrode pulverisation. Lijuan et al. [198] reported the synthesis of phenylalanine-functionalised graphene quantum dots (PF-GQD) from citric acid and phenylalanine via the pyrolysis method. Furthermore, a PF-GQD@SiNP composite was formed by coating PF-GQD on silicon nanoparticles and thermally annealed Ar/H_2_. With an increase in electrical conductivity, PF-GQD also helped to prevent direct contact of the silicon surface with electrolyte molecules. The composite electrodes exhibited excellent electrochemical performance in practical use for lithium-ion batteries. The specific capacities were reported as 4066 mA h g^−1^ at 50 mA g^−1^, 3796 mA h g^−1^ at 100 mA g^−1^, and 1820 mA h g^−1^ at 1000 mA g^−1^. The capacity endured 3068 mA h g^−1^ at 100 mA g^−1^ after 100 cycles. Additionally, alanine-functionalised graphene quantum dots (AF-GQD) were synthesised, and the results showed that the electrochemical performance of PF-GQD@SiNP was better than AF-GQD@SiNP due to the presence of benzene rings at the edges of graphene sheets, which resulted in wider and finer energy levels of electrons and well-defined steric structure of PF-GQDs. This report suggests an environmentally friendly, economical, and easy method for preparing silicon-based anodic materials for high-performance Li-ion batteries.

## 6. Conclusions and Future Aspects

In the coming era, CQDs and GQDs have immense potential due to their diverse optical, chemical, and physical properties in the field of optoelectronics, lasers, and light-emitting diodes. Furthermore, for energy storage, they are widely preferred materials for different ion-based batteries, supercapacitors, solar cells, advanced LEDs, etc. In this article, we have focused on and introduced the recent developments in the different simple fabrication techniques for CQDs and GQDs based on top-down and bottom-up approaches, along with other chemical routes derived from simply available raw materials. We have focused more on applications in the various kinds of light-emitting diodes and energy storage sectors due to their beneficial properties, such as low toxicity, simple fabrication, high efficiency, tunable emission range, higher stability, and promising optoelectronic properties. The fabrication route is a crucial step for high-quality quantum dots with desired properties. Additionally, in the future, a detailed analysis may be adapted to differentiate GQDs and CQDs based on their physical and chemical properties, as GQDs exhibit different physicochemical properties due to their varying shape, size, doping, and presence of various functional groups based on the synthesis route adopted. Therefore, a clear roadmap for GQDs based on their structure and composition with specific applications is still a topic of research. The current challenges limiting CQDs and GQDs in industrial and practical use include the low photoluminescence quantum yield (PLQY%) and production yield (generally around 30% by weight). Future studies may focus on simplifying the fabrication process using an eco-friendly and green synthesis route, with higher quantum yield and quantity with excellent fluorescent properties and detailed mechanisms resulting in realising large-scale production. Currently, the poor device performance of carbon/graphene-based LEDs is limiting the industrial production of future LED-based AR/VR devices. Carbon dot-based electroluminescent LEDs are still in the very early stages and need special attention in the near future. As CQDs possess size, shape, surface functional groups, and heteroatom doping-dependent physical and optical properties, controlling the size and morphology may be an important area for future studies. Additionally, the photoelectric conversion mechanism of carbon dot-based LEDs and luminescent mechanisms are still unclear and need to be explored shortly. Furthermore, GQDs have shown potential applications in light-emitting diodes, but still, the area is underexposed. Heteroatom-doped GQDs are an upcoming challenge that needs to be addressed in the near future to enhance the efficiency of various energy storage devices.

We firmly believe that addressing the above challenges will lead to the large-scale production of CQDs/GQDs having a potential application in future foldable, small-factor light-emitting diodes and several other fields, especially in the development of 5G/6G networks along with AI, which makes these fluorescent materials a potential candidate for next-generation LED technology with new innovations in device optimisation and applications based on carbon dots and practical use in day-to-day life in the near future.

## Figures and Tables

**Table 1:** Sodium and lithium-ion battery performance.

Battery and Method	Precursor	Material	Product	Cycle Stability	Specific Capacity (mA hg^−1^)	Ref
Na-ion(Hydrothermal)	Graphene oxide	GF aided VO2@GQD nanoarray as working electrode	GQD with size 5 nm	88% 1500 cycles	306 at 1/3 C	[188]
Na-ion(Hydrothermal)	Graphene oxide and melamine	Nitrogen doped GQDs as an anode	N-GQD	66% after 500 cycles	215 at 50	[187]
Li-ion(Hydrothermal)	Citric acid andL-cysteine	Lithium Titanate/N,S-GQDs as an anode	N,S GQD with size 4 to 7 nm	96.9% 2000 cycle	169 at 0.1 C	[182]
Li-ion(Hydrothermal)	Graphene oxide	GF aided VO 2@GQD nanoarray asworking electrode	GQD	94%1500 cycle	-	[188]
Li-ion(Hydrothermal)	Pyrene	NiO/GQDs-COOH electrode	GQDs-COOH	1081 mA hg^−1^ 250 cycles	1334 at 100	[184]
Li-ion(Hydrothermal)	Citric acid	GQDs based SiAl(NP-SiAl/GQDs)anode for lithium storage	GQD	52.6% 120 cycles	2507 at 200	[185]

## Data Availability

Data will be made available on request.
